# Greenhouse Gas Emissions from Asphalt Pavement Construction: A Case Study in China

**DOI:** 10.3390/ijerph13030351

**Published:** 2016-03-22

**Authors:** Feng Ma, Aimin Sha, Ruiyu Lin, Yue Huang, Chao Wang

**Affiliations:** 1 Key Laboratory Special Area Highway Engineering of Ministry of Education , Chang’an University, Xi’an 710064, China; amspaper@126.com (A.S.); ruiyu333@sina.com (R.L.); chaowangchd@126.com (C.W.); 2 School of Civil Engineering, Liverpool John Moores University, Peter Jost Enterprise Centre, Byrom Street, Liverpool L3 3AF, UK; Y.Huang@ljmu.ac.uk

**Keywords:** asphalt pavements, greenhouse gas, environmental impacts, construction process

## Abstract

In China, the construction of asphalt pavement has a significant impact on the environment, and energy use and greenhouse gas (GHG) emissions from asphalt pavement construction have been receiving increasing attention in recent years. At present, there is no universal criterion for the evaluation of GHG emissions in asphalt pavement construction. This paper proposes to define the system boundaries for GHG emissions from asphalt pavement by using a process-based life cycle assessment method. A method for evaluating GHG emissions from asphalt pavement construction is suggested. The paper reports a case study of GHG emissions from a typical asphalt pavement construction project in China. The results show that the greenhouse gas emissions from the mixture mixing phase are the highest, and account for about 54% of the total amount. The second highest GHG emission phase is the production of raw materials. For GHG emissions of cement stabilized base/subbase, the production of raw materials emits the most, about 98%. The GHG emission for cement production alone is about 92%. The results indicate that any measures to reduce GHG emissions from asphalt pavement construction should be focused on the raw materials manufacturing stage. If the raw materials production phase is excluded, the measures to reduce GHG emissions should be aimed at the mixture mixing phase.

## 1. Introduction

As the Chinese highway system continues to grow in mileage and traffic volume, it is important to construct highways sustainably and with low environmental impact. In China, the highway network is 4.5 million km in length, wherein the length of expressways is 111,900 km. For expressways, asphalt pavement is predominantly used, accounting for over 90%, compared to cement concrete pavement. The asphalt pavement is composed of aggregate, cement, and asphalt binders. The manufacture of the raw materials and construction of asphalt pavements consumes a lot of energy and emits large quantities of greenhouse gases (GHGs). 

Since the first expressway was built in China in the 1990s, the total expressway length has increased quickly up to the current 111,900 km by the end of 2014. Some 7500 km of expressway were built in 2014. Due to the increasing use of asphalt highways in China, the rapid growth of energy consumption and GHG emissions from its construction has caused public concern, making it necessary to assess the related environment impacts. However, there is a lack of suitable evaluation criteria and benchmark figures in China for GHG emissions generated from asphalt pavement construction. 

## 2. Literature Review

Horvath *et al.* studied the environment impacts of pavements made of asphalt and steel-reinforced concrete by a life cycle inventory analysis based on publicly available data [1]. They found that asphalt pavement appears to have higher energy input, lower ore and fertilizer input requirements, and lower toxic emissions, but generates higher amount of hazardous waste in comparison with steel-reinforced concrete pavement. 

Kim *et al.* conducted a series of studies on the GHG emissions from road construction projects. They established the framework method for the estimation of GHG emissions based on the data for a pavement project at the planning phase. The framework was applied to 23 typical highway construction projects in the Republic of Korea [2]. The project also studied the GHG emissions from onsite equipment usage during road construction, and summarized the eight major GHG-producing activities during the construction [3,4].

Hong *et al.* analyzed the GHG emissions during the construction of a building in China in an extended system boundary by using detailed onsite process data [5]. In the building process of infrastructure for urban highways, construction materials, building operations and transportation are found to be the main elements related to energy consumption and GHG emissions [6,7,8,9,10]. 

Santero and Horvath researched the global warming potential of pavement by dividing the construction into eight components: materials extraction and production, transportation, onsite equipment, traffic delays, carbonation, lighting, albedo, and rolling resistance. The ranges of potential impact for each component were calculated and compared. The results covered both the variability of pavements and uncertainty in the values. Two ranges were determined: a probable range of values based on the best estimates and an extreme range of value based on outlying data and less likely scenarios [11].

In 2010, greenhouse gas emissions in the United States totaled nearly 6.8 billon tons of CO_2_ equivalents. Of this total, the transportation sector was responsible for more than 1.8 billion tons of emissions, or 27.1% of total GHG. The transportation sector is the single greatest contributor of CO_2_ to the earth’s atmosphere in the U.S. and accounts for about 31.1% of all CO_2_ emissions [12].

Huang developed a life cycle assessment model for construction and maintenance of asphalt pavement. Details are presented on both the methodology and data acquisition in the U.K. The model is applied to an asphalt pavement project comparing the environmental impact of virgin aggregate, waste glass, incinerator bottom ash and recycled asphalt pavements [13,14].

The energy consumption and environmental impacts of asphalt and reinforced concrete pavement (materials and construction) were researched by Zapata [15]. According to the study, the main consumption of energy from extraction to asphalt placement occurs during the mixing and drying of aggregate (48%) for the pavement. Moreover, the production of bitumen accounts for about 40% of the total energy consumption. 

The GHG emissions related to highways and vehicles have attracted the interest of researchers for the last 20 years [16,17,18,19,20,21,22]. The highway construction industry plays an important role in economic development, but is also a main source of carbon emissions. The GHG emissions from aggregate heating, bitumen refinery, and mixture mixing phase have been evaluated [17]. The total emissions are estimated by adding those from different processes of construction by different project types, such as subgrade, pavement, bridge, and tunnels [22].

Globally, there are several tools like LEEDS and GreenRoad in the U.S. and CEEQUAL and asPECT in the U.K., available to measure the CO_2_ or sustainability. Other tools are available in Australia and Germany. In addition, many studies have evaluated the GHG emissions related to the highway infrastructure construction. The oversea evaluation methods and resources are mostly based on local data that are not representative of the Chinese circumstances. Moreover, some methods and software are commercial products, not available for academic research. At the present, the main challenge in the study of environmental impacts of asphalt pavement in China is a lack of project validated data and sector approved methods of life cycle carbon analysis. This study focuses on the GHG emissions of asphalt pavement construction. The process is divided into the production of raw materials, the mixture mixing, mixture transportation, paving, and rolling of the asphalt mixture. Moreover, at high temperature, the GHG emissions from asphalt mixture are included.

## 3. Materials and Methods

### 3.1. Evaluation System Boundary

**Figure 1:**
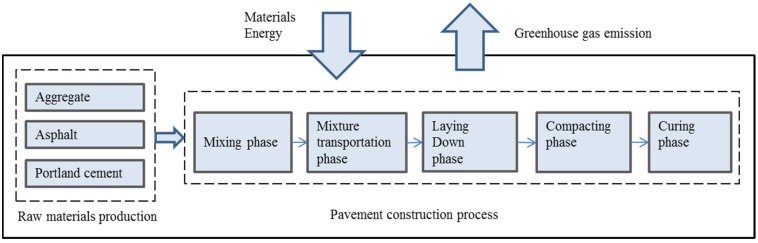
Evaluation system boundary of GHG emissions for asphalt pavement construction.

This study focuses on GHG emission from the raw material production and pavement construction, as shown in Figure 1. This includes the emissions from all components of the asphalt pavement, including the extraction of raw materials, transport and the onsite placement of the asphalt pavement. The first process includes the production of aggregates, asphalt and Portland cement. The second process includes mixing, transportation, paving, compacting and curing phase of cement stabilized aggregate course.

According to the Kyoto Protocol, there are six maim greenhouse gases, namely carbon dioxide (CO_2_), methane (CH_4_), nitrous oxide (N_2_O), hydrofluorocarbons (HFCs), perfluorocarbons (PHCs) and sulphur hexafluoride (SF_6_) [23]. Since HFCs, PFCs and SF6 are not commonly present in the asphalt pavement construction process, this study only focuses on three types of GHG: CO_2_, CH_4_ and N_2_O.

### 3.2. Evaluation Method

The simplest expression of a GHG account (*E_GHG_*) is the product of activity data (AD) and emission factor (EF), shown as Equation (1) below.

(1) $$E_{GHG}=AD\times EF$$

While carbon dioxide is the GHG of greatest concern, there are several other GHGs. As the global warming potential (GWP) of these GHGs varies, a group of conversion coefficients are established to convert the emission of a specific GHG into carbon dioxide equivalents (CO_2_e). In this context, GWP is the integral of the global warming effect of a GHG compared with that of CO_2_ in the same time interval, commonly using a time horizon of 100 years. The 100 year GWPs of CO_2_, CH_4_ and N_2_O are 1, 23 and 296 respectively [24]. Therefore:

(2) $$CO_{2}e=AD\times EF\times GWP$$

The carbon account of asphalt pavement is the sum of all relevant emission sources, so the final expression of the asphalt pavement’s carbon footprint can be given by Equation (3):

(3) $$E_{GHG}=\overset{n}{\underset{i=1}{\sum }}{\left(CO_{2}e\right)}_{i}=\overset{n}{\underset{i=1}{\sum }}(AD_{i}\times EF_{i}\times GWP_{i})$$

where (*CO*_2_e)*_i_* means the carbon account from the single procedure in asphalt pavement, and the other indexes are the same as in Equation (2).

### 3.3. Energy Consumption Method

Most pavement construction activities are carried out with heavy machinery and equipment. The GHG emissions of asphalt pavement consists of those from the machines and equipment used in the construction process. It is calculated by multiplying the energy consumption data (AD) by the emission factor (EF) of each energy type, fuel or electricity, as shown in Equation (4). Emission factors for typical construction equipment and machinery from Chinese statistics [25] are adapted to this study.

(4) $$E_{GHG}=AD_{fuel\;or\;electricity}\times EF$$

### 3.4. Field Measured Method

**Figure 2:**
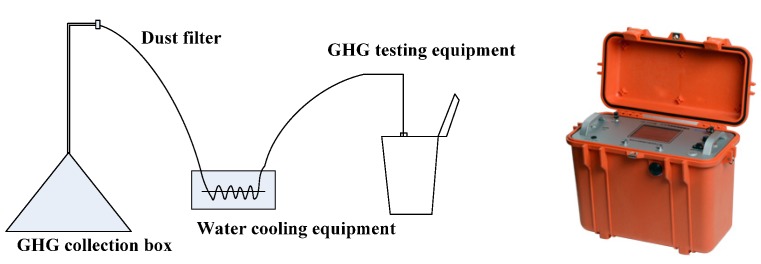
Schematic of GHG testing equipment and testing equipment.

At a high temperature, asphalt mixtures emit GHGs and bitumen fumes. This is in addition to the GHG emissions from the asphalt mixture during the mixing, transportation, laying and compacting processes. This is evaluated following Equation (5):

(5) $$E_{GHG}=C\times L$$

where C is measured concentration, mg/m^3^; L is gas volume, m^3^. The measurement method and equipment, shown in Figure 2, was developed by Chang’an University. The GHG sensor is able to measure CO_2_, CH_4_ and N_2_O; the units are ppm. The equipment is designed based on infrared spectroscopy theory. The CO_2_, CH_4_ and N_2_O sensor operates using non-dispersive infrared gas detection. The sensor comprises a tungsten light source, an optical path for gas diffusion, a semiconductor temperature sensor and a signal exchange electronic circuit, and the thermoelectric infrared detection component. The GHG collection box is cylinder-shaped, with a radius of 18 cm.

Very few studies in the past have concentrated on the non-energy related emissions, such as from the bitumen fuming. This is mainly due to the fact that: (1) field data are difficult to collect, and (2) the variance in the properties of different bitumens makes the results debatable and difficult to compare. The GHG mass in specific volume is converted, according to Equation (6), from the ppm results:

(6) $$C=\frac{C^{\prime }\times M}{22.4}$$

where, C is the GHG concentration in mg/m^3^; C′ is the GHG concentration, in ppm; M is the molecular weight; 22.4 is the average molar volume of air under standard conditions (0 °C and 101.325 kPa). During the onsite construction process, the GHG output is affected by the temperature of the mixture, emission time and surface area of the mixture. For example, in the mixture transportation phase, the mixture is typically piled up in a truck to about 1 m height. The surface area is relatively small. However, during the laying and compacting phase, the surface area of the same mixture is much larger, so different coefficients are estimated for the different construction phases. The time interval of GHG emissions is assumed for the onsite construction phase. Then, the amount of GHG is calculated for the mixture mixing phase, mixture transportation phase , laying and compacting phase.

### 3.5. Data Analysis Method

For evaluating GHG emissions from the production of raw materials, data from literature and industry databases for cement, asphalt and aggregate materials are chosen in this evaluation system [25]. Based on the field data and pavement geometric size, the amount of materials is calculated. According to published data on machinery efficiency and construction characteristics in China, the working time is calculated. The Chinese highway engineering quota method is used to determine the working time. The data on machinery efficiency is obtained from the rated value of manufacturers. Finally, some data is gathered from field investigations.

## 4. Case Study

A case study of GHG evaluation is provided for a Chinese asphalt pavement construction project. Results can be valuable to researchers who compare the CO_2_e output of asphalt pavement projects and seek the causes of any differences, as well to designers and contractors who want to benchmark their design and construction options.

### 4.1. Description of Project

**Figure 3:**
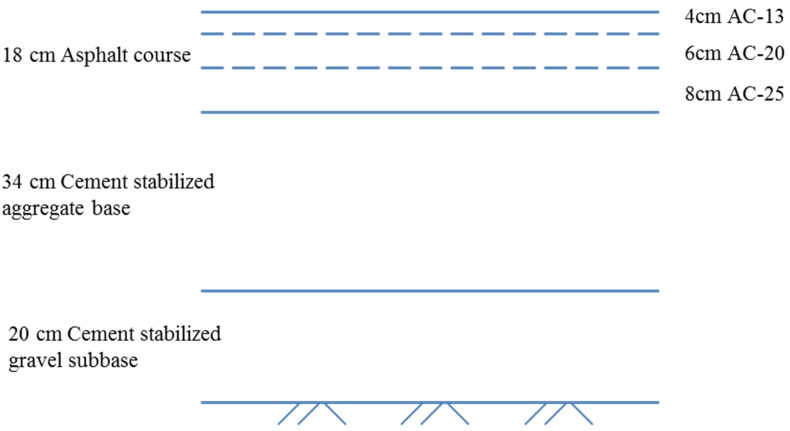
Schematic diagram of the asphalt pavement structure.

The case study is carried out on a typical four lane asphalt pavement of 20 km in length. The depth of the pavement structure is 72 cm. The width of the pavement is 28 m. The lane width is 3.75 m each. The widths of the hard shoulder and verge are 3.5 m and 0.75 m, respectively. At present, a semi-rigid base, which is usually produced from cement stabilized aggregate or gravel, is commonly used in China. The pavement structure, which consists of a cement stabilized aggregate base and an asphalt mixture surface is shown in Figure 3. The mixing plant is set up temporarily during the project. The average distance between the mixing plant and the construction site is 10 km. 

Polymer-modified asphalt is used in the surface layer. Asphalt of penetration grade 70# is used in the other two layers. Emulsified asphalt is used in seal coat, prime coat and bond coat. The binder of the base and subbase is Portland cement. The GHG emissions from transportation between the quarry and the asphalt mixing site are taken into account. The average distance is used to calculate the GHG. The pavement raw material quantities can be calculated from the volume because the quantities are proportional to the cross-section area and length. The density of asphalt mixture is 2.42 g/cm^3^, and the density of cement stabilized aggregate is 2.20 g/cm^3^. Based on the geometry of the pavement structure and material density, the total pavement materials quantities are calculated, as shown in Table 1. The usage of equipment is calculated based on the Chinese specifications and data collected from construction sites.

### 4.2. Evaluation of GHG Emissions for Raw Material Production

In this phase, the environmental burdens come from aggregate acquisition and processing. Energy consumption for aggregate production includes the rock blasting, quarrying, hauling, crushing and screening. The GHG emissions, such as CO_2_, CH_4_, NO and SF_6_, are mainly from the energy consumption of machinery and explosives. In an aggregate quarry, the main energy consumption corresponds to electricity and diesel used by the plants and equipment. The electricity is produced from other energy sources such as coal and gas. At present, coal is the primary energy source used in China to produce electricity. The working hours are calculated from the quantities of raw materials divided by capacity of the equipment. Then the amount of electricity and diesel is calculated. The GHG emissions from consumption of electricity and diesel are then be calculated following Equation (1). According to Equation (2), the quantities of CH_4_ and N_2_O are converted to CO_2_ equivalents, with global warming potentials of 23 and 296, respectively. The total CO_2_e is shown in Table 2.

The production of asphalt binder is a complex process. Moreover, there are different sources of GHG emissions during the process. The European bitumen association has presented data with an energy consumption of 510 MJ/t and CO_2_ 174.244 kg/t, NO_X_ 0.770 kg/t and CH_4_ 0.595 kg/t emissions for straight-run asphalt binder production [26]. Chinese asphalt production mainly uses the oxidation method and solvent method based on the production of vacuum distilled residue. CO_2_, CH_4_ and N_2_O are generated from the transportation vehicles, machinery and equipment during the process. Fossil fuel and electricity are consumed in the process. Due to the differences in manufacturing technology, the GHG emissions are derived from electricity consumption and fossil fuel combustion. The CO_2_e is computed from the amount of the two types of energy based on average data for China [25]. Energy consumption for asphalt binder production includes crude oil extraction, transport, and refining. The GHG emissions of pavement courses are shown in Table 3 and Table 4.

In this study, the Chinese reference emission data for cement products is used. Dry production is a typical method in China. In this process, every 1t of cement produced consumes 97.4 kWh of electricity, 116 kg of coal and 0.2 L of diesel. At the same time, it produces GHG emissions including 781 kg of CO_2_, 1.53 kg of N_2_O and 3.414 kg of CH_4_ [25]. 

For cement stabilized aggregate base and cement stabilized gravel subbase, the raw materials are the aggregate and the cement. The aggregate production data was already calculated in the previous section. There are abundant data in the literature for estimating the GHG emissions from the cement production phase.

### 4.3. Evaluating of GHG Emissions for Construction Process

#### 4.3.1. Construction Process of Asphalt Mixture Course

For an asphalt surface, the total GHG emissions in CO_2_ equivalent come from the production of raw materials, mixing, mixture transportation, paving, and rolling of asphalt mixture. In this process, the GHG emissions were calculated based on the energy consumption of machinery and manufacturing plants. For a semi-rigid base/subbase, the total GHG emissions in CO_2_e come from the production of raw materials, mixing, mixture transportation, paving, and rolling of cement bound mixture. 

**Figure 4:**
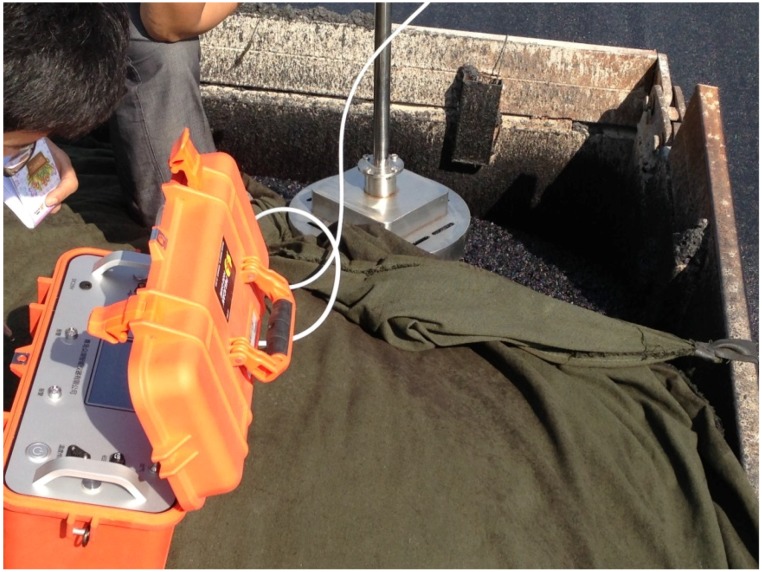
The field testing of GHG emission in transportation phase.

**Figure 5:**
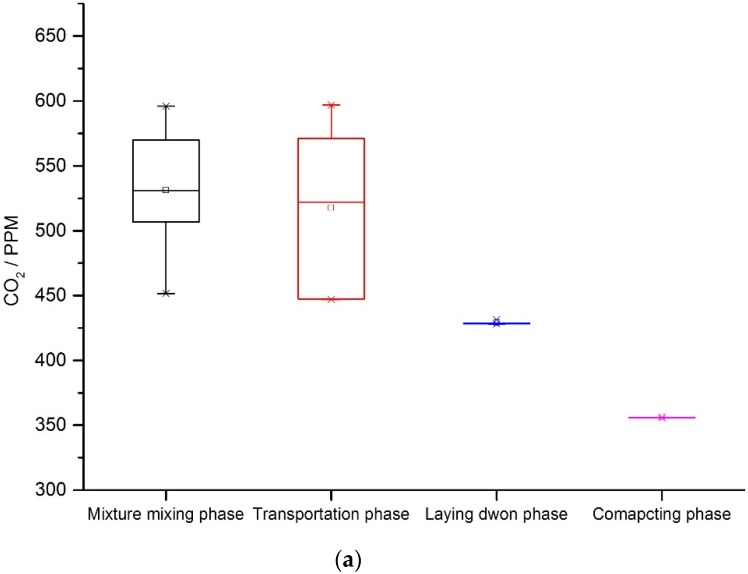

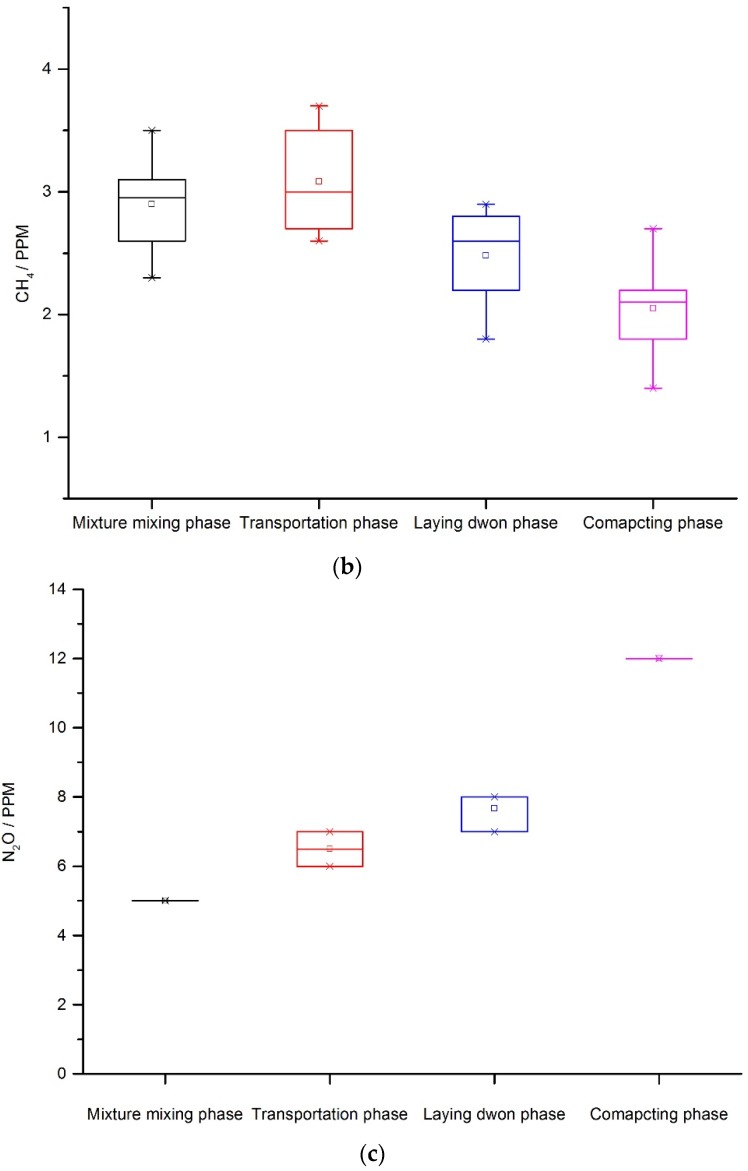
(**a**) CO_2_ concentration from onsite construction process; (**b**) CH_4_ concentration from onsite construction process; (**c**) N_2_O concentration from onsite construction process.

For the mixing phase, the GHG emissions come from energy consumption of mixing equipment, fuel combustion in heating the aggregate and asphalt binder, and gas emissions from the hot asphalt mixture. The control system, mixing cylinder, material transferring component, sieving and weighting components are operated by electricity. The aggregate is fed into the cold material silo by the loader machine. Fossil fuel is consumed in the aggregate and asphalt heating system. Fossil fuel and electricity are consumed in the mixing phase. CO_2_, CH_4_, N_2_O are generated in the mixing phase. For the transportation phase, the environmental impacts are due to the emissions released by the engines of the transport vehicles. All materials were assumed to be hauled by heavy-duty vehicles. The average fuel consumption and transportation distances were determined from literature and field data, respectively. For the asphalt mixture laying phase, the GHG comes from energy consumption of the paver and gas emission from hot asphalt mixture. For the compacting phase, the GHG comes from diesel consumption of the rollers, and gas emission from hot asphalt mixture. Figure 4 illustrates the field test results for the transportation phase. The test results are shown in the Figure 5a–c. 

The GHG emissions from fuming asphalt mixture are shown in Table 5. The total CO_2_e is calculated, by Equation (2), to be 5652.54 kg. 

The total GHG emissions for construction of asphalt course are shown in Table 6.

#### 4.3.2. Construction Process of Cement Stabilized Aggregate Courses

For the construction process of cement stabilized aggregate courses, in the mixture mixing phase, transportation phase, laying down phase, and compaction phase, the GHG evaluation is similar to that for the asphalt courses. For the curing phase, the GHGs come from the fuel consumption of the trucks used for spraying water. The fuel consumption is calculated based on working time of trucks and amounts of materials. The CO_2_e is evaluated following Equations (1) and (2). The total GHG emissions for construction of cement stabilized aggregate base is shown in Table 7.

## 5. Results and Discussion

### 5.1. Analysis of the Total GHG Emissions

For the 20 km long asphalt pavement case study, the total GHG emissions of construction include asphalt concrete courses and cement stabilized aggregate base/subbase. The construction process includes the mixture mixing phase, transportation phase, laying phase, compacting phase, and curing phase for cement stabilized aggregate. The total CO_2_e emission of the 20 km asphalt pavement construction is 52,264,916.06 kg. The CO_2_e emission of asphalt course is 9,123,898.74 kg, or about 17.46% of the former. The CO_2_e emission of cement stabilized aggregate course is 43,141,017.32 kg, or about 82.54%. The latter amount is about 4.7 times the former one. The GHG emissions from fuming asphalt mixture are shown in Table 5. The total CO_2_e is calculated, by Equation (2), to be 5652.54 kg. 

**Figure 6:**
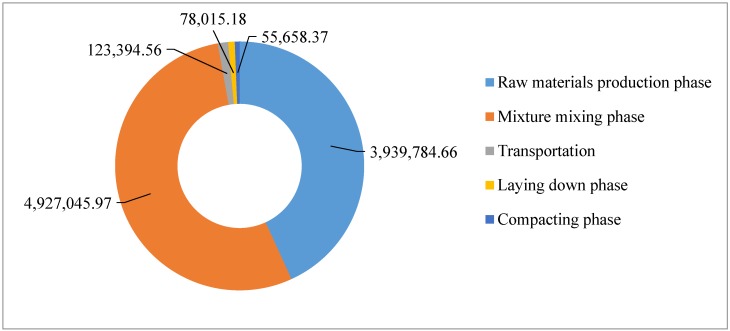
CO2e emission of asphalt course construction (unit: kg).

The total GHG emissions of asphalt construction are shown in Figure 6. It can be seen that 97.19% of the total GHG emissions are due to the mixture mixing phase and raw materials production phase, wherein 54.01% are from the mixture mixing phase, and 43.18% are from the raw materials production phase. About 1.35% of the total GHG emissions are due to the raw material and mixture transportation phase. Only 0.86% and 0.61% of the total GHG emissions are due to laying phase and compacting phase.

**Figure 7:**
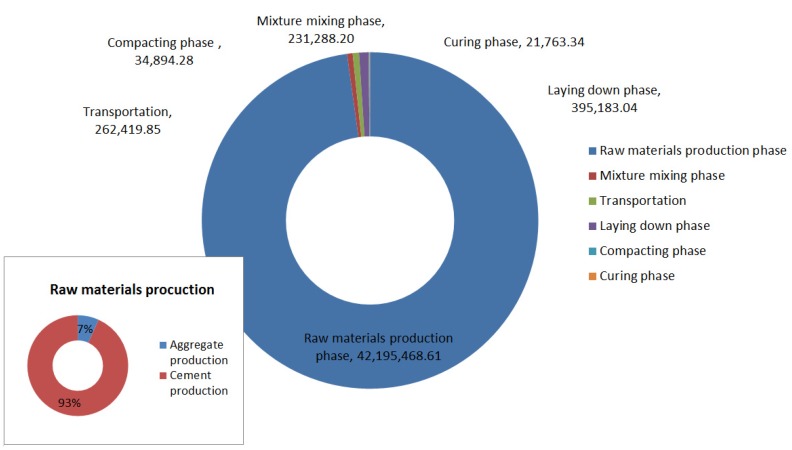
CO_2_e emission of cement-stabilized aggregate base and subbase construction (unit: kg).

The cement-stabilized aggregate base is similar to the cement-stabilized gravel subbase, so the environmental impact of the two layers is presented together. The total GHG emissions of the cement-stabilized aggregate course is shown in Figure 7. The GHG emissions of raw material production are the largest, and account for about 97.81%, in which 91.14% are from the cement production, and 6.86% are from the aggregate production. Only 0.54% of total GHG emissions are due to the mixture mixing, while 0.61% of the total GHG emissions are due to the raw material and mixture transportation, 0.92% are due to the laying phase, and 0.08% and 0.05% of the total GHG emissions are due to the compacting phase and the curing phase, respectively.

**Figure 8:**
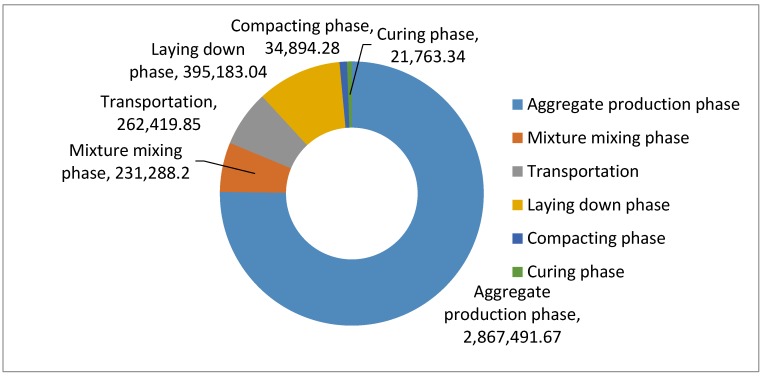
CO_2_e emission of cement-stabilized aggregate course excluding the cement production phase (unit: kg).

Cement is a typical construction material, which is widely used in the building and civil sector. Nowadays, a great amount of cement is being produced for China’s rapid urbanization. The road pavement construction is an important part within the sector. Figure 8 shows the total GHG emissions of the cement-stabilized aggregate course, excluding the cement production phase. From Figure 8, 75.20% of the GHG emissions are due to the aggregate production, while 6.07% are due to the mixture mixing, 6.88% are due to the raw material and mixture transportation, 10.36 are due to the laying phase and finally 0.92% and 0.57% of the total GHG emissions are due to the compacting and curing phase, respectively.

### 5.2. Analysis of GHG Emissions Excluding Raw Material Production Phase

As seen in Section 5.1, the raw materials production accounts for most of the total GHG emissions, where the cement, aggregate and asphalt binder production generate 88.27% of the total. Moreover, the asphalt mixture contributes to 9.43% of total GHG emissions which comes from heating the huge amount of aggregate and asphalt needed. The raw materials production phase is aligned with the current technology in China. 

**Figure 9:**
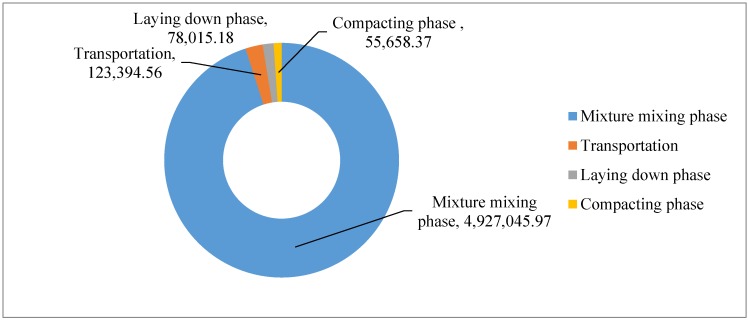
CO_2_e emission of different phases in asphalt course construction excluding raw materials production process (unit: kg).

For transportation sector managers and researchers, it is important to know that the GHG emissions are due to the different phases of asphalt pavement construction. The CO_2_e emissions of different phases in asphalt course construction excluding raw materials production are shown in Figure 9. About 95.04% of the GHG emissions are due to the mixture mixing phase, and 2.38% of the GHG emissions are due to the raw material and mixture transportation phase. Only 1.50% and 1.07% of the total GHG emissions are due to laying phase and compacting phase, respectively.

**Figure 10:**
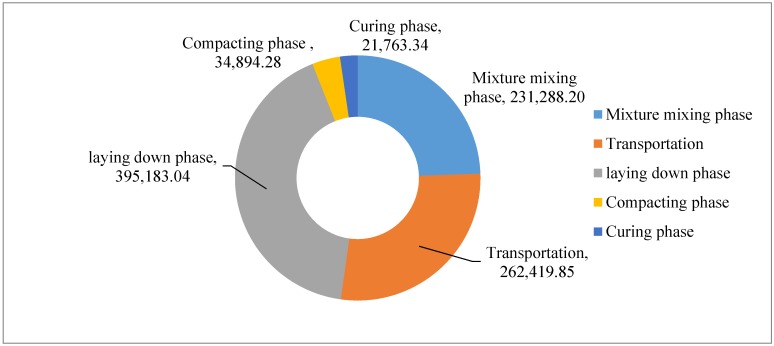
CO_2_e emission of different phases in cement-stabilized course construction excluding raw materials production process (unit: kg).

The GHG emissions of different phases in cement stabilized course construction excluding raw materials production are shown Figure 10. The total CO_2_e emissions are obviously small, *i.e.*, 945,548.71 kg. About 41.79% of the GHG emissions are due to the laying phase, whereas 27.75% are due to the raw material and mixture transportation phase, 24.46% are due to the mixture mixing phase and only 3.69% and 2.30% of the GHG emissions are due to the compacting and the curing phase.

### 5.3. Discussion and Recommendations

The results indicate that the raw materials production phase and the mixture mixing phase contribute to the most GHG emissions for the asphalt mixture course. Whether considering the cement production or not, the raw materials production phase contributes to the most GHG emissions for the cement stabilized aggregate courses. The use of raw materials with low GHG emissions and increasing the efficiency of asphalt mixing equipment are good starting points to reduce energy consumption and GHG emissions. The warm/cold asphalt mixture can be helpful to decrease the GHG emissions thanks to a lower mixing temperature. In addition, emulsion asphalt could be a good choice to reduce the environmental impact. The benefit of using reclaimed asphalt pavement (RAP) is the reduced extraction and production of virgin aggregates. 

Due to the large amount of cement used as raw material in the base and subbase, although the energy consumption of asphalt surface layer is high, its GHG emissions are relatively low. When the raw materials are not considered, the energy consumption of the asphalt surface layer is the largest, and the energy consumption and emissions of cement stabilized aggregate base/subbase are relatively small. 

Therefore, in the design of the asphalt surface courses, the main focus should be the control of energy consumption, the use of energy−efficient equipment and the optimization of the construction management. In the design of cement stabilized aggregate base/subbase, the main focus should be to decrease the GHG emissions from the raw materials production. The use of high efficiency and energy saving methods of cement production can achieve the purpose of energy saving and emission reduction.

The data in this case study are collected from the project and industry database in China. The data system related to GHG emissions of Chinese highways is still at an infant stage. On the other hand, the fast growth of new built highways in China calls for a bespoke model and robust data to measure and reduce the GHGs from the road sector. The evaluation of GHG emissions due to the highway maintenance will be a very important area for research in the years to come. 

## 6. Conclusions

Asphalt pavement construction has significant environmental impacts. Its GHG emissions are evaluated and calculated for a case study in China, including raw materials production, mixing, transportation, laying, compacting and curing phase. The total CO_2_e emission of the 20 km long asphalt pavement construction project is 52,264,916.06 kg. 

For the asphalt pavement construction, the mixture mixing phase generates the largest amount of GHG emissions, accounting for 54% of the total. The raw material production accounts for 43% of total GHG emissions. For cement-stabilized aggregate base/subbase, the largest portion of GHG is emitted in the raw materials (cement and aggregates) production phase, accounting for about 98% of total emissions, wherein the cement production emissions alone accounted for 92% of the total emissions of raw materials production phase.

For the asphalt mixture course construction, the use of energy-saving and efficient equipment is recommended to decrease the GHG emissions. For cement stabilized aggregate course, the improvement of cement and aggregate production will help decrease the GHG emissions. If the raw material production is excluded, the use of energy-saving and efficient equipment for laying, mixing and transportation is recommended to decrease the GHG emissions.

## Figures and Tables

**Table 1:** Mass of raw materials in pavement structure (units: t).

Asphalt Course			Cement Stabilized Aggregate Base		Cement Stabilized Gravel Subbase	
Asphalt binder	Aggregate	Mineral powder	Cement	Aggregate	Cement	Aggregate
8270.21	183,139.07	12,819.74	16,298.99	325,979.79	9008.61	180,172.20

**Table 2:** GHG emissions of mineral aggregate production.

Structure	Mass of Aggregate/t	Energy Consumption/MJ	CO_2_e/kg
Aggregate in asphalt course	183,139.07	11,410,751.00	1,069,398.55
Aggregate in cement stabilized base	325,979.79	19,825,054.00	1,857,495.72
Aggregate in cement stabilized subbase	180,172.20	10,771,278.00	1,009,995.95
Mineral powder in asphalt course	12,819.74	998,006.97	94,760.11
Total	702,110.80	43,005,089.97	4,031,650.33

**Table 3:** GHG emissions of asphalt courses.

Structure	Asphalt Type	Mass of Binder (t)	Energy Consumption (MJ)	CO_2_e (kg)
Top surface course	Modified asphalt	2015.29	4,816,642.63	533,369.53
Middle and nether layers	Base asphalt	6254.92	14,146,081.61	1,579,145.03
Seal coat	Emulsified asphalt	2573.01	5,954,312.55	662,431.95
Total		10,843.22	24,917,036.80	2,774,946.51

**Table 4:** GHG emissions of cement stabilized courses.

Cement Stabilized Aggregate Base			Cement Stabilized Gravel Subbase		
Mass of cement (t)	Energy consumption (MJ)	CO_2_e (kg)	Mass of cement (t)	Energy consumption (MJ)	CO_2_e (kg)
16,298.99	325,979.79	27,967,146.59	9008.61	38,295,090	11,360,830.35

**Table 5:** The GHG emissions from fuming asphalt mixture (units: kg).

Construction Process	CO_2_	CH_4_	N_2_O
Mixture mixing phase	261.60	0.52	2.46
Mixture transportation phase	85.02	0.19	1.07
Laying down phase	211.22	0.45	3.79
Compacting phase	263.04	0.56	8.87

**Table 6:** GHG emissions for construction of asphalt course.

CO_2_e Emissions/kg			
Mixture mixing phase	Transportation phase	Laying down phase	Compacting phase
4,927,045.97	123,394.56	78,015.18	55,658.37

**Table 7:** GHG emissions for construction of cement stabilized aggregate base.

CO_2_e Emissions (kg)				
Mixture mixing phase	Transportation phase	Laying down phase	Compacting phase	Curing phase
231,288.20	262,419.51	395,183.04	34,894.28	21,763.34
